# EBV-specific T-cell immunity: relevance for multiple sclerosis

**DOI:** 10.3389/fimmu.2024.1509927

**Published:** 2024-12-24

**Authors:** Malina Behrens, Manuel Comabella, Jan D. Lünemann

**Affiliations:** ^1^ Department of Neurology with Institute of Translational Neurology, University Hospital Münster, Münster, Germany; ^2^ Servei de Neurologia-Neuroimmunologia, Centre d’Esclerosi Múltiple de Catalunya (Cemcat), Institut de Recerca Vall d’Hebron (VHIR), Hospital Universitari Vall d’Hebron, Universitat Autònoma de Barcelona, Vall d’Hebron University Hospital, Barcelona, Spain

**Keywords:** multiple sclerosis, Epstein-Barr virus (EBV), T cell immunity, autoimmune disease, neuroinflammation

## Abstract

Genetic and environmental factors jointly determine the susceptibility to develop multiple sclerosis (MS). Improvements in the design of epidemiological studies have helped to identify consistent environmental risk associations such as the increased susceptibility for MS following Epstein–Barr virus (EBV) infection, while biological mechanisms that drive the association between EBV and MS remain incompletely understood. An increased and broadened repertoire of antibody and T-cell immune responses to EBV-encoded antigens, especially to the dominant CD4^+^ T-cell EBV nuclear antigen 1 (EBNA1), is consistently observed in patients with MS, indicating that protective EBV-specific immune responses are deregulated in MS and potentially contribute to disease development. Exploitation of B-cell trajectories by EBV infection might promote survival of autoreactive B-cell species and proinflammatory B:T-cell interactions. In this review article, we illustrate evidence for a causal role of EBV infection in MS, discuss how EBV-targeting adaptive immune responses potentially modulate disease susceptibility and progression, and provide future perspectives on how novel model systems could be utilized to better define the role of EBV and viral pathogens in MS. Insights gained from these studies might facilitate the development of prevention strategies and more effective treatments for MS.

## Introduction

Multiple sclerosis (MS), afflicting more than 2.5 million people worldwide, is the most common chronic inflammatory, demyelinating disease of the central nervous system (CNS) (1). The disease occurs in young people with a complex predisposing genetic background and is likely to be triggered by an inciting environmental insult. Evidence from both experimental and clinical observations suggests that an autoimmune dysregulation of the adaptive immune system is at the core of MS. This is supported by genome-wide association studies, which revealed multiple associations with immune-system-related gene variants, most importantly the HLA-DR15 haplotype (2) as well as focal MS lesions being thought to be elicited by the infiltration of immune cells, including T cells, B cells, and myeloid cells, into the CNS parenchyma (3). Additionally, T and B cells isolated from CNS lesional tissue and the cerebrospinal fluid (CSF) of MS patients have been found to be largely derived from clonal expansion (4, 5); intrathecally produced oligoclonal antibodies present in the CSF show evidence for antigen-dependent affinity maturation (6), and a variety of immunotherapies targeting lymphocyte survival, function, or migration have been shown to be beneficial in treating MS (7). Lastly, the experimental animal model most closely mimicking MS, collectively termed experimental autoimmune encephalomyelitis (EAE), is largely driven by autoimmune T cells (8).

Infections have long been suspected as one of the culprits in the development of MS. While epidemiological data increasingly support this connection, immunological and virological evidence remains inconclusive. As a result, a wide range of potential pathogens has been investigated as possible triggers for MS, including measles, rabies, herpes simplex, herpes zoster, human herpesvirus, rubella, mumps, scrapie-like agents, paramyxoviruses, coronaviruses, canine distemper, Marek’s Semliki forest virus, various animal and human retroviruses, human T-cell leukemia virus type I, and Epstein–Barr virus (9). Almost universally, close examination of these candidates failed to verify a clear association, resulting in the notion that the postulates of Koch have to be met unequivocally to establish a clear pathogenic role of an infectious agent in MS. Yet, during the past 20 years, high-quality epidemiological, serological, and immunological studies in large cohorts of clinically well-defined patients and appropriate controls consistently report a strong association of Epstein–Barr virus (EBV) infection and immune responses to EBV with the development of MS.

## EBV life cycle and associated diseases

**Figure 1:**
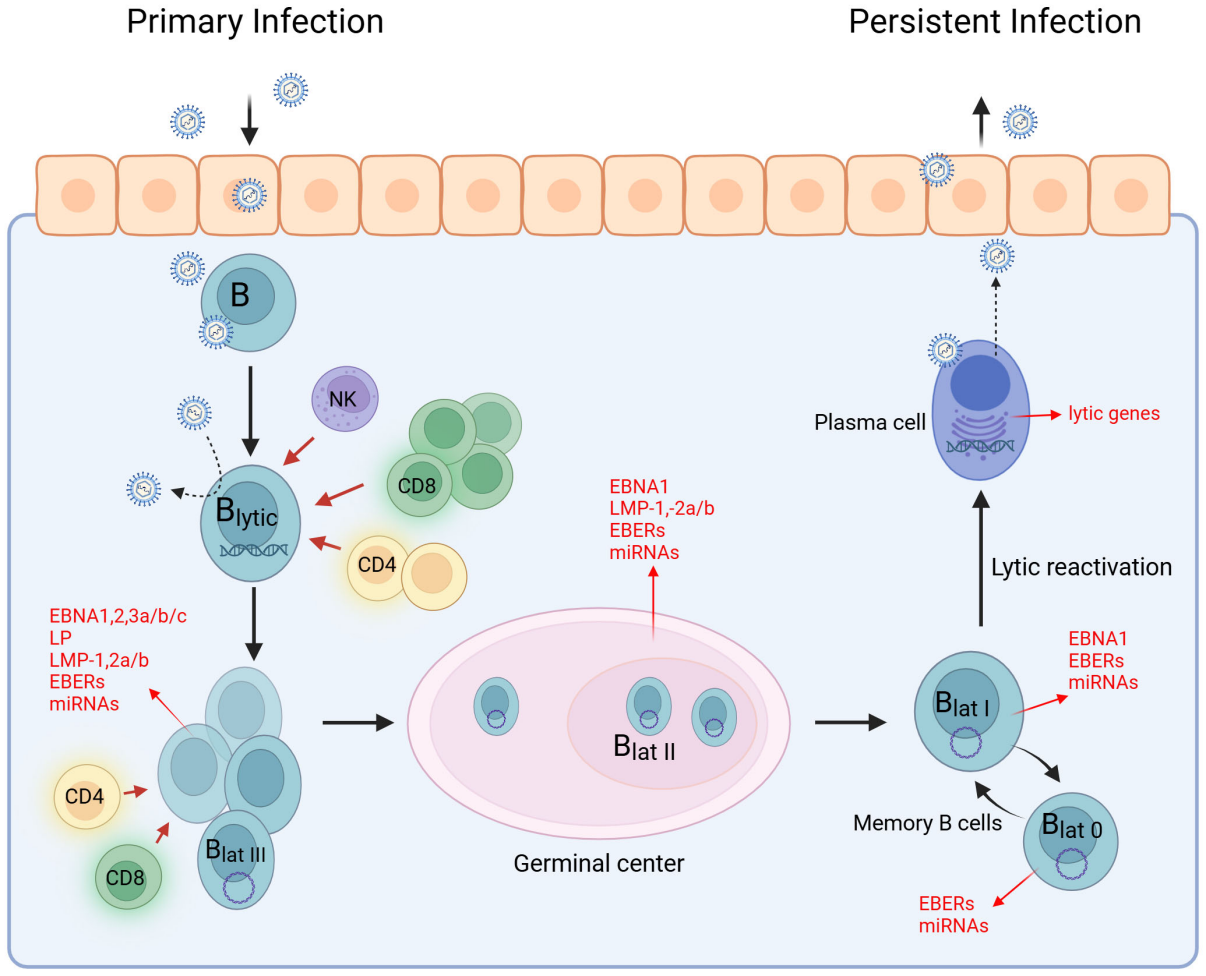
EBV life cycle. The life cycle of EBV can be divided into the lytic and latent phases. EBV infects epithelial and B cells in the oropharynx, leading to a high viral load. During the latent phase, EBV spreads by promoting B-cell proliferation while expressing all latency antigens (latency III). In the germinal center reaction, the expression of latency antigens is downregulated to EBNA1, LMP1, and LMP2 (latency II). B cells develop into centroblasts and then centrocytes. Centrocytes leave the germinal center, differentiate into memory B cells, and circulate in the peripheral blood (latency I). Some transition to a stage known as latency 0, where no EBV antigen expression occurs. The EBV-infected memory B cells serve as a reservoir for the virus and can differentiate into plasma cells. EBV occasionally reactivates and re-enters the lytic cycle. Created with BioRender.com.

The Epstein–Barr virus (EBV) is a globally prevalent virus, which, despite its often harmless initial infection, can have lifelong effects and is associated with a variety of serious diseases like cancer and autoimmune diseases. EBV was discovered in 1964 in Burkitt lymphoma (BL) as the first human tumor virus (10), and since then, EBV has been found in a variety of other tumor types (11–13). As a member of the γ-herpesvirus family, EBV establishes a lifelong latent infection with periodic reactivation, enabling efficient transmission to new susceptible individuals (14–16). Consequently, more than 90% of the world’s population is infected with the virus. Primary infection is often asymptomatic and occurs at a young age (17). However, infection during teenage years or young adulthood can lead to infectious mononucleosis (IM), characterized by symptoms such as fever, severe fatigue, swollen lymph nodes, and sore throat (18, 19). EBV is primarily transmitted via saliva (20) and infects human B cells and epithelial cells in the oropharynx (21, 22). In both cell types, the virus can replicate efficiently and shuttle mainly between these cell types (23). Oral replication leads to a high viral load during the lytic phase, characterized by a marked increase in CD8^+^ T cells, which are crucial for eliminating infected cells, with support from CD4^+^ T cells and NK cells (24–26) (**Figure 1**). EBV then enters its first latent stage and spreads by inducing B-cell proliferation. During the viral gene expression program known as latency III, all latency proteins of the virus are produced. This results in the activation, proliferation, and resistance to cell death of latency III-infected B cells (27–29). A robust T-cell response targets latency III-infected cells, which decrease in number much more rapidly than cells infected in the lytic phase (30). Following the latency III phase, the expression of latent proteins is reduced to EBNA1, LMP1, and LMP2 upon entry into the germinal center reaction (28). This transition to latency II likely supports the survival of EBV-infected B cells within the germinal center reaction and enables access to the memory B-cell pool where EBV remains without viral protein expression, representing latency 0 (31, 32). During homeostatic proliferation, only EBNA1 is expressed, a state known as latency I (16). EBV-infected memory cells can recirculate from the blood into lymphoid organs, and the lytic cycle can be periodically reactivated. The exact triggers for this reactivation are not yet fully understood, but it is believed that stimulation of the B-cell receptor by a cognate antigen may play a role in initiating this process (33, 34).

## Association of infectious mononucleosis with MS susceptibility

A connection between EBV and MS was first suspected over 40 years ago when researchers found that antibodies against the EBV capsid antigen (VCA) were significantly elevated in individuals with MS compared to healthy controls (35). Since then, growing evidence has reinforced the idea that EBV infection is linked to a higher risk of developing MS (36–39). Moreover, the risk of MS is two- to three-fold higher among individuals with a history of infectious mononucleosis (IM), a symptomatic primary infection with EBV (40). Both MS and IM are rare in populations, where infections occur at an early age, following a latitude gradient. Primary infections with EBV in adolescence or adulthood are more common in countries with higher socioeconomic status and hygiene standards (41). Additionally, the prevalence and incidence rates of MS are higher in developing countries. These observations led to the assumption that a late infection with EBV, which is often characterized with IM, could be an important risk factor for MS. However, it has been hypothesized that IM and MS are not causally related but may share the same etiology: high level of hygiene during childhood could be a common factor contributing to both IM and MS. This hypothesis is based on the assumption that high hygiene standards may increase the susceptibility to autoimmune diseases (37). However, several studies challenge this notion by suggesting a direct link between MS and EBV infection. The hygiene hypothesis posits that individuals who lack EBV infection and were subjected to high sanitary standards and limited infections during early life may have an elevated risk of developing MS. Indeed, according to a model from Loosen 2022 that takes into account the link between EBV infection and MS, the risk of MS is highest in people who first became infected with EBV in adolescence or later in life and is in the medium range in people who became infected in early childhood (40). However, the noteworthy discovery is that the probability of developing MS is almost negligible in people who have not been infected with EBV (40). This strongly suggests a causal relationship between IM and MS. In other studies, a similar observation has been reported (39), but the mechanisms by which EBV and specifically IM contribute to an increased risk of MS are not yet fully understood. It is speculated that the intense immune activation during IM (unlike asymptomatic EBV infection) may result in enhanced priming of self-reactive immune cells by molecular mimicry and bystander activation (42). Self-reactive cells could promote local inflammation and induce damage in brain tissue such as central neurons and their myelin sheaths (43, 44). Consequently, this increased activation during IM could potentially elevate the risk of developing MS.

## Serological studies

The potential link between EBV and the development of MS has been under suspicion for an extended period (38, 45, 46). However, despite numerous epidemiologic studies identifying EBV as a potential etiologic risk factor (47–50), establishing causality has proven challenging. This difficulty arises from the challenge of determining whether individuals who develop MS after EBV infection would have developed MS without prior infection. The task is further complicated by the fact that more than 90% of the global adult population is EBV seropositive, but the majority do not develop MS (51, 52). Secondly, the long interval between the biologically measurable onset of the disease and its clinical manifestation adds another layer of complexity to this determination.

Remarkably, longitudinal studies analyzing serum samples obtained from healthy adults before the manifestation of MS have revealed a significant elevation in EBV antibodies several years before the initial onset of MS symptoms (47, 48). Notably, the most robust association was identified with EBNA1-specific and EBNA2-specific IgG (49), suggesting a potential link between elevated antibody levels and the subsequent development of MS (50). The increase in EBNA1 antibody titers was not only detected in blood samples but was also seen in the CSF of a large proportion of MS patients and could therefore potentially contribute to MS pathology (53). IgG1 was identified as the most frequently detected isotype, contributing to enhanced humoral immunity (54). A recent study from 2022 by Bjornevik et al. provided the clearest epidemiological evidence to date that EBV infection is a prerequisite for the development of MS. In this study, they identified MS cases in a cohort from more than 10 million active US army personnel between 1993 and 2013 (39). A total of 801 individuals who developed MS during active duty and 1,566 controls were included in the study. Three blood samples collected prior to the onset of MS were available for serum analysis and to assess the EBV infection status. As a result, the study revealed a 32% increase in the risk of developing MS following EBV infection. Initially, 35 of the current MS patients tested negative for EBV at the baseline sample collection, but all, except for one, subsequently became infected with EBV and underwent seroconversion before the onset of the disease. Still, for this isolated case, it cannot be excluded that an EBV infection occurred after the last blood collection (3 months before disease onset), the individual did not undergo seroconversion in response to the infection, or there might have been a possibility of misdiagnosis. An infection with the cytomegalovirus, another herpes virus family member (55) with infectious epidemiology similar to that of EBV (56–58), did not result in an increased risk of MS even after seroconversion (39). This finding further strengthens the selective association between EBV and MS. Neurofilament light chain (sNfL), a biomarker of ongoing neuroaxonal degeneration (59), but not disease-specific, was employed to elucidate the temporal relation between EBV infection and MS. The levels of sNfL increased after EBV infection in individuals who later developed MS, but there was no corresponding increase after infection in the control group. Before EBV infection and around the time of infection, the sNfL levels of MS patients did not differ from those observed in the control group. In another study conducted in 2015 using samples from U.S. military personnel, it was further demonstrated that individuals with higher anti-EBNA antibody titers (≥320) were 36 times more likely to develop MS compared to those with a titer of less than 20 (47). These titers can therefore represent the first reliable serological biomarkers for the risk of MS together with sNfL levels. Bjornevik et al. further used an unbiased assay (VirScan) to analyze antibody responses to a broad range of human viruses and found that only the response to EBV was significantly elevated in MS patients compared to controls (39). Moreover, Cortese et al. observed that while MS patients display a stronger overall immune response to the EBV peptidome, they recognized the same EBV epitopes as controls. However, the antibody response to EBNA1 turned out to be the strongest serologic risk factor (60). This reinforces the assumption of a specific interaction between EBV and MS that is distinct from other viral diseases (39).

## Deregulated EBV-specific T-cell immunity in MS

The initiation of EBV-specific immune control is probably mediated by dendritic cells presenting EBV antigens from infected B cells, and then it centers around strong memory CD4^+^ and CD8^+^ T-cell responses, whereby the CD4^+^ T cells maintain EBV-specific Th1 immunity, and both CD4^+^ and CD8^+^ T cells target EBV-infected cells directly (61). CD8^+^ T cells expand dramatically during acute infection, with up to 25% to 50% of all CD8^+^ T cells being directed against individual EBV lytic antigens in certain patients with the symptomatic primary EBV infection IM. During persistent EBV infection, both lytic and latent EBV antigen-specific CD8^+^ T cells can be maintained at frequencies of up to 1% to 5% of peripheral blood CD8^+^ T cells. CD4^+^ T cells are thought to orchestrate virus-specific immune responses and are crucial for the priming and maintenance of CD8^+^ T cells (61). The functional differentiation of virus-specific CD4^+^ T cells is crucial for efficient humoral or cell-mediated immune responses. Th1 responses, which are characterized by the secretion of the antiviral cytokine gamma interferon, are more protective against viral infections and support the generation of virus-specific CD8^+^ T cells, which are the effectors of cell-mediated adaptive immunity (62). Even during primary infection in IM patients, EBV-specific CD4^+^ T cells never reach the high frequencies of EBV-specific CD8^+^ T cells in peripheral blood (63). Virus-specific CD4^+^ T cells reach only 1/10 of the frequency of their EBV-specific CD8^+^ T-cell counterparts during primary and persistent infection, and it has become evident that CD4^+^ T cells target a different set of latent EBV antigens than CD8^+^ T cells (64). An increased risk of MS is associated with human leukocyte antigen (HLA)-DRB1*15:01, thereby implicating CD4^+^ T cells as potent effectors in the disease.

**Figure 2:**
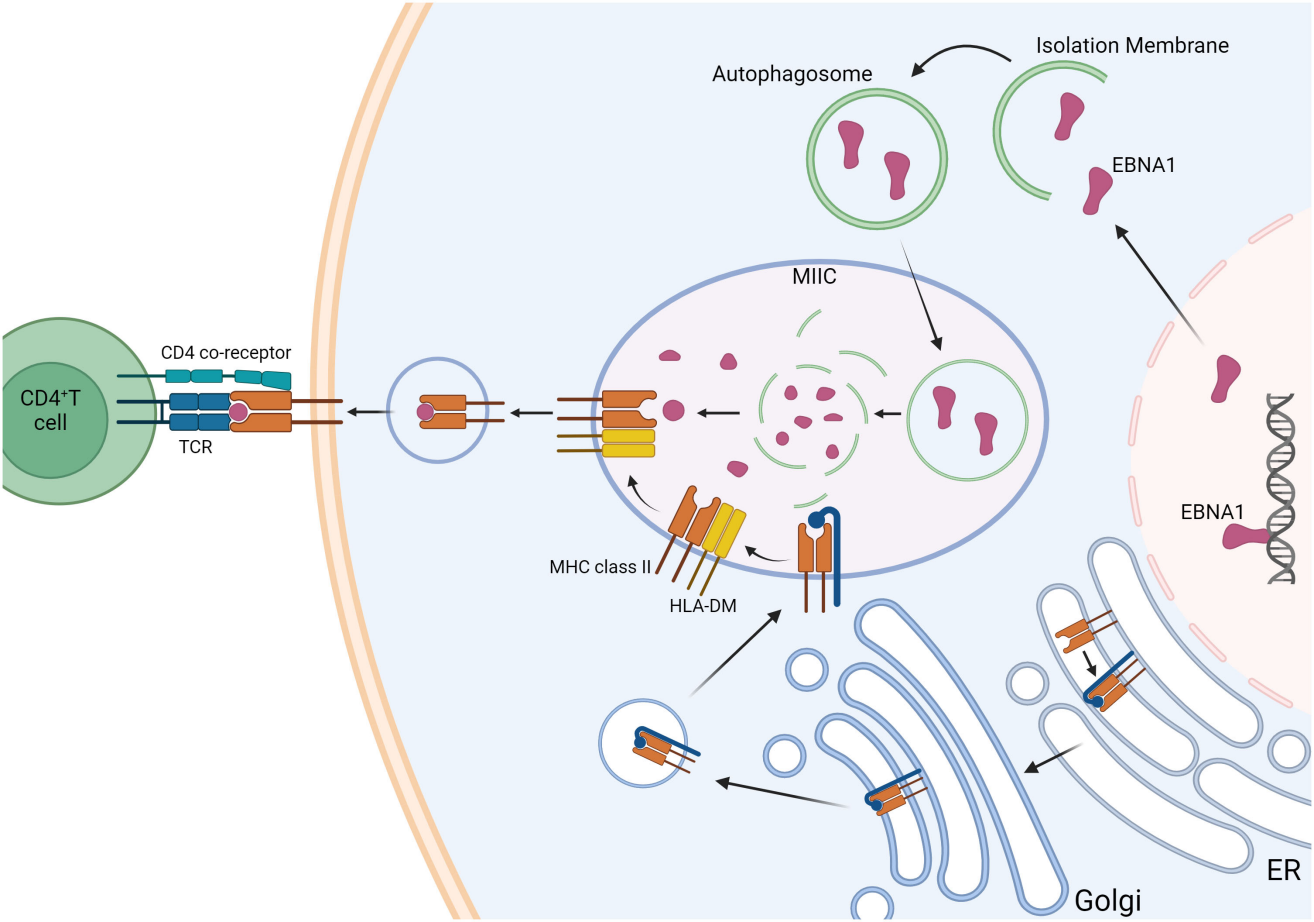
Autophagy-associated EBNA1 presentation pathway. EBNA1 is a nuclear antigen presented on MHC class II molecules. Its presentation on MHC class I is inhibited by blocking proteasome-dependent protein degradation. Intracellular antigens like EBNA1 can be processed via autophagy, allowing them to enter the pathway for endogenous MHC class II presentation. Lysosomal proteases degrade EBNA1, and in the MHC class II-loading compartments (MIICs), antigen peptides are loaded onto MHC class II molecules. This process is facilitated by the peptide-loading chaperone HLA-DM, which promotes the exchange of peptides on MHC class II molecules and enhances their stability before they are transported to the cell surface for recognition by CD4^+^ T cells. MHC class II molecules are synthesized in the endoplasmic reticulum (ER) and associate with a glycoprotein called invariant chain (blue). This invariant chain contains a targeting signal that directs the MHC class II molecules to the endosomal pathway. Created with BioRender.com.

EBNA1 was shown to be consistently recognized by CD4^+^ T cells of healthy EBV carriers and evoked responses more frequently than any other latent EBV antigen. EBNA1-specific CD4^+^ T cells are Th1 in function and are considered to be a crucial component of EBV-specific immune control. The characteristic glycine-alanine repeat of EBNA1 inhibits its proteasomal degradation (65) but does allow MHC class II loading via autophagy-mediated pathways and the recognition of EBNA1-expressing targets (**Figure 2**) (66). EBNA1 is therefore the only EBV antigen consistently expressed in proliferating cells with latent EBV infection in healthy virus carriers and represents a key target antigen for CD4^+^ T-cell-mediated immune control mechanisms of EBV infection in healthy individuals. MS patients show increased responses and levels of activated HLA-DR-restricted CD4^+^ T cells that are specific for EBNA1. The enhanced response to EBNA1 is mediated by an expanded reservoir of EBNA1-specific central memory CD4^+^ Th1 precursors and Th1 polarized effector memory cells. Moreover, the CD4^+^ Th1 phenotype cross-reacts with myelin autoantigens and produces IFN-γ (54). It can be speculated that these autoreactive T helper cells could potentially activate autoreactive B cells, leading to the production of autoantibodies.

CD8^+^ T cells are important effector cells in controlling viral infections. Prolonged antigen stimulation, as in chronic viral infections, can lead to exhaustion of the CD8^+^ T cells and reduce their activity and function (67). A striking indication of the contribution of this T-cell subset is their presence in brain lesions of MS patients in even greater numbers compared to CD4^+^ T cells (68–70). MHC class I alleles are also associated with MS; for example, the HLA-A-030 allele increases the risk of developing the disease two-fold (71). Mainly effector memory T cells infiltrating the CSF of MS patients (72) and increased granzyme B levels were found during relapses suggesting a higher CD8^+^ T-cell-mediated cytotoxicity (73, 74). Moreover, CD8^+^ T cells expand in areas of EBV-infected cells in the brain and show elevated cytotoxic activity. It is suggested that this may also contribute to damage of tissue and potentially affect myelin sheaths (75). However, the precise impact of CD8^+^ T cells in association with EBV on MS remains to be fully elucidated.

In contrast to the pathology-promoting effects of CD8^+^ T cells in MS, Vietzen et al. recently found that EBV-specific, HLA-restricted CD8^+^ T cells may be important in killing EBV-infected autoreactive B cells because these specific cells exhibit a robust response against EBV-infected GlialCAM_370–389_-specific B cells. The authors postulate that greater activation and proliferation of these specific CD8^+^ T cells has a protective effect on MS pathology (76).

Not only adaptive immune cells can influence MS pathology but also innate immune cells; NK cells could have a non-negligible influence. NK cells are lymphocytes with cytotoxicity and cytokine-producing effector functions that can recognize and kill abnormal cells such as tumor cells and virus-infected cells. They identify and kill infected or tumor cells by recognizing specific ligands presented by MHC class I molecules on the surface of these target cells (77). Vietzen et al. found that certain NK cells exert a protective effect against the development of MS. NKG2C^+^ and NKG2D^+^ NK cells exhibit heightened activation against GlialCAM-specific cells, and increased levels of these NK cells were observed in healthy individuals with high EBNA-specific IgG. The levels of these specific NK cells can be influenced by the stabilization of HLA-E through UL40 variants of prior HCMV infections or *KLRC* variants of the cells. Inhibition of NKG2A^+^ NK cells, caused by cellular resistance mechanisms of EBV-infected autoreactive B cells, can potentially increase the risk of MS (76).

## How EBV-specific immune responses potentially interfere with MS pathogenesis

**Figure 3:**
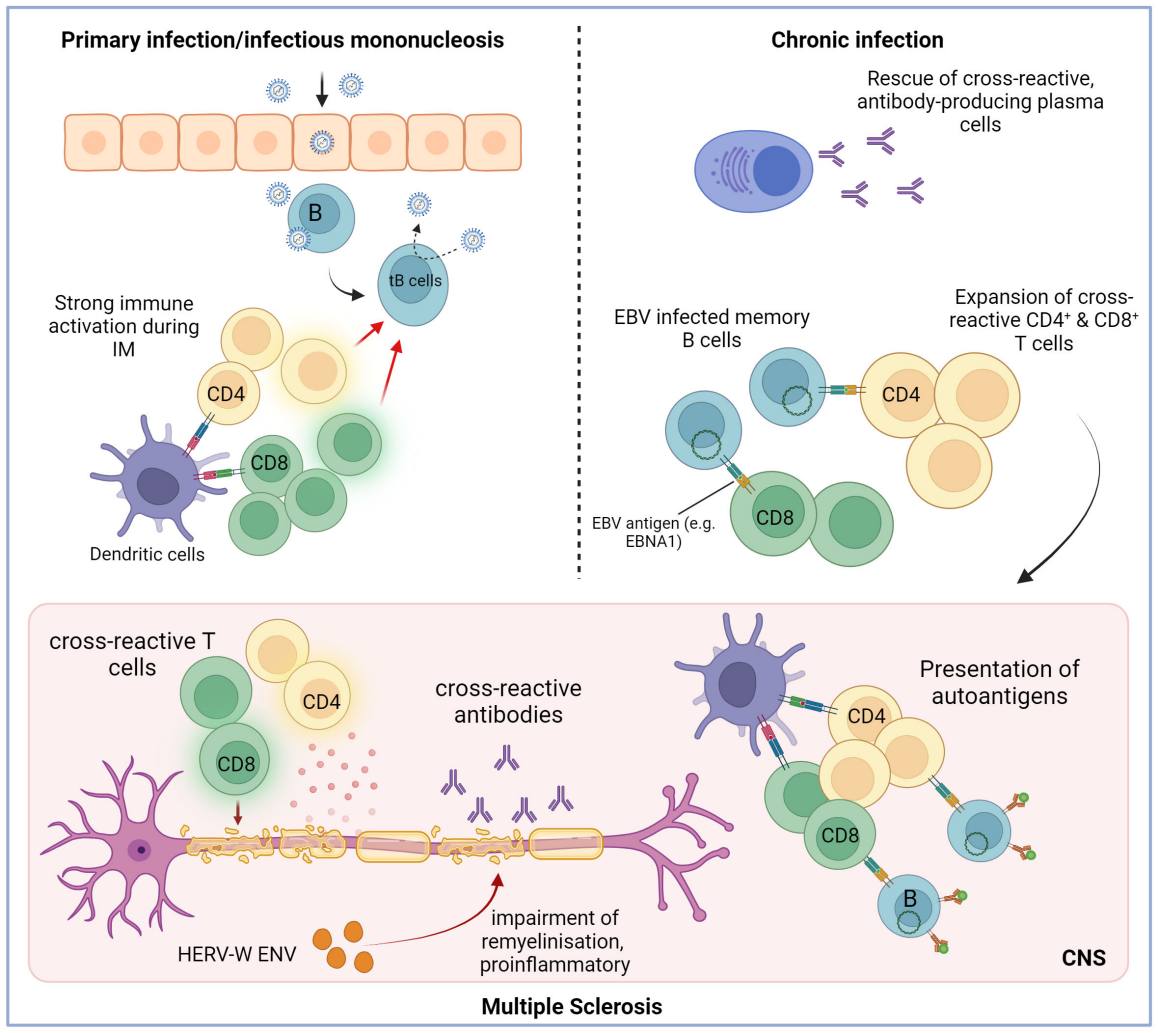
Potential mechanism through which Epstein–Barr virus infection may trigger the initiation of multiple sclerosis. The initial infection with EBV can trigger a robust immune response, often resulting in infectious mononucleosis (IM). It is a lytic infection that is mainly brought under control by EBV-specific CD8^+^ T cells but also by CD4^+^ T cells. The virus infects B cells and spreads by inducing a latent, growth-transforming infection in the B cells (tB cells: transformed B cells). The strong immune activation during the lytic infection could promote the activation and expansion of autoreactive and cross-reactive T and B cells. These self-reactive cells might be maintained due to constant antigen exposure and deficient immunoregulatory networks. Additionally, one potential role of EBV is to stimulate the proliferation and maturation of B cells while also preventing the apoptosis of autoreactive B cells and plasma cells that generate autoantibodies. Hence, it is conceivable that the activation of self-reactive T cells and the production of cross-reactive antibodies contribute to demyelination in MS. HERV-W ENV may drive the disease progression even further by inhibiting remyelination and promoting inflammation. Created with BioRender.com.

We propose four main possible scenarios that could explain the altered humoral and cell-mediated immune responses to EBV in patients with MS and the potential contribution of EBV to the pathogenesis of the disease (**Figure 3**). The scenarios are based on the assumption that host factors predisposing to MS, such as allelic variants of susceptibility genes, influence the immune response to EBV, and all scenarios could apply to other autoimmune diseases. It is important to point out that EBV might not only be a trigger for MS but could also contribute to disease progression after disease onset (78). This distinction is crucial since the latter scenario would offer opportunities to treat MS by targeting EBV infection (78). In addition to B-cell-directed depleting strategies ranging from conventional antibody-based CD20 treatment to specifically EBV-targeting synthetic cell-based approaches (79), which target the virus reservoir, direct antiviral treatments such as tenofovir (80) could also be employed.

### Protective immune responses to EBV initiate and sustain autoimmunity in MS

Strong innate immune activation during primary EBV infection might promote the activation and proliferation of autoreactive and polyspecific T and B cells, which recognize both autoantigens and viral antigens. MS patients show increased frequencies of CD4^+^ T cells specific for EBNA1 and higher activation states (81, 82). It is possible that continuous stimulation through cross-reactions with autoantigens (54), i.e., molecular mimicry, could maintain these cells and enhance EBV-specific CD4^+^ T-cell responses (**Figure 1**). Such specific cross-reactions between the central nervous system (CNS) and foreign antigens have been previously identified (83–85). In particular, T cells specific to EBNA1 have been shown to react to a mixed myelin antigen pool (54), and CD4^+^ T-cell cross-reactivity between EBNA1 and alpha-crystallin B was discovered in mouse experiments (86). Another study reported broader EBV-specific TCR repertoires in MS patients, even between monozygotic twins, discordant for MS (87). Beyond this, cross-reactivity is not limited to T cells; autoantibodies targeting specific regions of EBNA1 have also been identified in MS patients. The amino acid region aa385–440 especially plays an important role in molecular mimicry and is associated with MS risk (88). Lanz et al. found out that a monoclonal antibody present in the CSF of 20%–25% of MS patients, which binds EBNA1_AA386–405_ with high affinity, cross-reacts with the glial cellular adhesion molecule GlialCAM. Co-immunization with this epitope triggered a strong B-cell response against GlialCAM in mice and thus induced autoimmune demyelination (89). Antibodies targeting EBNA1_401–420_ can also bind alpha-crystallin B peptides containing the homologous motif (90). Elevated autoantibody reactivity to anoctamin 2 (ANO2), a Ca²⁺-activated chloride channel, has been detected in MS patients, with anti-ANO2 antibody levels being 5.3-fold higher compared to controls (84). Reciprocal blocking experiments showed that EBNA1 peptides (aa431 to 440) cross-reacted with anoctamin 2 (aa140 to 149) (86). The persistent activation of autoreactive T cells and the production of cross-reactive antibodies may not only initiate demyelination but also exacerbate disease severity by driving chronic inflammation and tissue damage, ultimately contributing to the progression of neurodegeneration in multiple sclerosis. Nevertheless, it is important to note that while various cross-reactive antibodies have been identified, they are present in only a subset of patients, and their precise role in the disease remains poorly understood.

### EBV assists in the maintenance of autoreactive B cells

B-cell-depleting therapies in MS show rapid and potent efficacy and strengthen the hypothesis that B cells may contribute to the pathogenesis of MS (91). Additionally, oligoclonal bands (OCBs), used as a diagnostic feature of MS (92), are elevated in MS patients, indicate the intrathecal B and plasma cell activity, and correlate with increased disease activity and disability (93). Some of the intrathecal antibodies specific to EBV were identified to cross-react with CNS antigens in MS patients (89). It is suspected that in MS, autoreactive B cells infiltrate the CNS, where they contribute to axonal injury and demyelination. In line with this, Pender’s hypothesis postulates that chronically EBV-infected autoreactive B cells accumulate in the brain and can produce pathogenic autoantibodies, which may also provide costimulatory signals for autoreactive T cells and thus prevent their apoptosis (94). This accumulation of EBV-infected autoreactive B cells in MS is proposed to be driven by a genetic defect that impairs the elimination of EBV-infected B cells by CD8^+^ T cells, which would normally keep EBV under control (94). Key predictions of this hypothesis have been subsequently verified, including the presence of EBV-infected B cells in the brain and meninges of MS patients (75, 95–97), decreased CD8^+^ T-cell immunity to EBV in MS (98), and the therapeutic efficacy of B-cell-depleting therapies such as ocrelizumab (99, 100). Nevertheless, it should be mentioned that some studies have not consistently detected EBV-positive B cells in the CSF or CNS lesions of patients with MS (101–103). These discrepancies may partly result from technical limitations, such as difficulties in the localization of EBV-infected cells or limitations of postmortem tissue analyses. Autoreactive B cells are normally controlled and neutralized by various tolerance checkpoints during development and differentiation (104). EBV persists in memory B cells and has an impact on their differentiation and function. Latent EBV B-cell infection provides B-cell survival advantages to escape the mechanisms of B-cell tolerance and could thus support the survival of autoreactive B cells in MS patients (105, 106). In fact, in mice transgenic for the latent EBV membrane protein 2 (LMP2) with targeted expression in their B-cell compartment, peripheral B cells were found to escape neutralization in the germinal center despite defective B-cell receptor expression (107).

Disease-relevant autoreactive B cells in MS could be specific for myelin antigen, while some studies indicate that CSF B cells contribute to MS pathogenesis by presenting self-antigens in the form of idiotope peptides from their B-cell receptors (BCRs) (108–110). These peptides, particularly from the complementarity-determining region (CDR3), are predicted to have a high affinity for HLA-DR molecules and are processed by cathepsins, which enables their presentation on HLA class II molecules (109). The presentation of these specific BCR-derived peptides activates memory CD4^+^ T cells in MS patients (108) Moreover, the CDR3 sequences of CSF B cells from MS patients contain more T-cell-exposed motifs, which have a higher potential to evade immune tolerance and activate CD4^+^ T cells compared to B cells from patients with other inflammatory neurological disorders (109). Therefore, it can be speculated that idiotope-driven T–B-cell interactions are involved in the pathogenesis of MS by driving its chronicity. Despite the promising insights, studies on idiotope-driven T–B-cell interactions in MS are still limited. Further research, especially *in-vitro* and *in-vivo* studies, is needed to strengthen our understanding of how T-cell activation through idiotopes contributes to the disease.

Dysregulated intrinsic B-cell control of EBV gene expression can lead to a proinflammatory, pathogenic B-cell phenotype. Spontaneous lymphoblastoid cell lines (SLCLs) derived from B cells of MS patients during active disease exhibited significantly higher EBV lytic gene expression than SLCLs from patients with stable disease or healthy controls. Moreover, host gene expression analysis revealed activation of pathways linked to hypercytokinemia and interferon signaling in MS-derived SLCLs and an upregulation of the transcription factor forkhead box protein 1 (FOXP1), which is important for maintaining EBV lytic gene expression, altogether indicating that a population of EBV-positive B cells, which fail to control EBV latency, may contribute to MS pathogenesis by driving B- and T-cell inflammation, particularly during active disease (111).

Thus, EBV gene products might stimulate cross-reactive autoimmune B cells directly or increase their survival after infection. Chronic stimulation of infected autoreactive B cells by autoantigens could, in turn, drive the replication of the virus and trigger enhanced, potentially pathogenic T-cell responses.

### EBV transactivates other viral elements that are cytotoxic to CNS-resident cells

Approximately 8% of the human genome consists of sequences of viral origin, the human endogenous retroviruses (HERVs). HERVs are probably the remnants of an ancient germ cell infection by exogenous retroviruses and have long been suspected of being involved in the development of autoimmune diseases (112). Various studies reported higher HERV expression in MS patients and an association between HERVs, particularly the HERV-W family and MS (112, 113). The envelope protein (ENV) of HERV-W is suspected of contributing to neurodegeneration in MS. In the progressive stages of the disease, HERV-W ENV leads to a degenerative phenotype in microglial cells, driving them toward a close spatial association with myelinated axons (114). Additionally, in HERV-W ENV-stimulated myelinated co-cultures, microglia were found to structurally damage myelinated axons. HERV-W ENV also negatively affects oligodendroglial precursor cell (OPC) differentiation and remyelination (114). Moreover, the HERV-W ENV glycoprotein, also known as syncytin-1, is upregulated in glial cells within acute demyelinating lesions in MS patients and induces redox reactant release that mediates the death of oligodendrocytes (115). EBV has been demonstrated to possess the ability to trigger the expression of diverse HERVs (116, 117). Consequently, EBV-mediated transactivation of HERVs, which may lead to axonal injury in the CNS, could potentially play a role in the development of MS. EBV itself can be reactivated by the human herpesvirus 6 (HHV-6). It has been observed that infection of EBV-positive cells with HHV-6 significantly increases the expression of EBV lytic cycle proteins, including the Zebra antigen and early antigens. This interaction suggests that HHV-6 directly stimulates EBV replication, potentially contributing to EBV-related diseases such as multiple sclerosis (118).

### Altered immune responses to EBV as a non-pathogenic epiphenomenon

It is possible that altered responses to the Epstein-Barr virus (EBV) result from specific host factors that increase susceptibility to autoimmune diseases, yet they may not directly contribute to the pathogenesis of MS. Since the virus latently persists in B cells, an effective cell-mediated immune response is required to limit the replication of the virus. Defects in the immune functions, not necessarily attributed to a causative role of EBV, could involve increased stimulation of the B-cell compartment by autoantigens. This stimulates increased EBV replication, leading to elevated viral load and consequently triggering enhanced antiviral immune responses. Alternatively, autoimmunity due to dysregulated regulatory T cells could also lead to increased EBV-specific immune responses without them being responsible for MS pathogenesis. In particular, lupus patients exhibit an unusually high number of EBV-infected B cells in their blood, which express abnormal levels of viral lytic (BZLF1) and latent (latency membrane proteins 1 and 2a) genes (119). It is hypothesized that this observation may be based on defects in the immune functions of systemic lupus erythematosus (SLE) patients without the necessity to assert or exclude EBV’s causal role in SLE pathogenesis.

## Concluding remarks and future directions

EBV has been a leading candidate trigger for several autoimmune diseases since the initial description of increased EBV-specific antibody titers in patients with SLE in 1971 (120). In principle, the long coevolution and the intertwined relationship of EBV with the human immune system, particularly the virus’ influence on B-cell biology and the requirement for a strong protective T-cell response, are compatible with both a pathogenic and an epiphenomenal function of EBV in autoimmune diseases. Strong evidence for a causal role of EBV in MS development stems from recent, large epidemiological studies (39).

Nonetheless, despite all the studies suggesting that EBV infection is a necessary condition for MS based on the virtually universal seroprevalence of EBV among MS patients, the mechanisms by which EBV triggers or maintains MS development remain poorly understood. In this context, further research dissecting in depth the interactions between the virus and its host is needed for a better understanding of how EBV infection can lead to the development of MS. We need to develop new *in-vitro* and *in-vivo* models that allow us to gain insight into the interactions between the main players of MS, namely, the immune cells, CNS cells, and EBV. *In-vitro* models based on reprogramming of blood cells into neural cells and neural organoids that can be co-cultured with immune cells become increasingly available. Such organoids, derived from induced pluripotent stem cells (iPSCs) of MS patients, may provide a valuable *in-vitro* system to investigate how patient-specific genetic backgrounds influence neural cell function and their interactions with EBV in a controlled microenvironment (121–123). Similarly, humanized mouse models, which are engineered to have a reconstituted human immune system, provide a unique platform to study the interplay between EBV and the human immune response *in vivo* (124). These models can help explore the mechanisms of how EBV infection affects brain function and tissue injury and also can help study specific B-cell or T-cell subpopulations directly involved in disease development. We also need to gain more knowledge on how coinfection of EBV with other viruses such as cytomegalovirus, which has been shown to reduce MS risk (125, 126) and positively influence prognostic disease outcomes (127, 128), modulates the interaction between EBV and the immune system. Finally, we need to identify reliable biomarkers that allow for monitoring virus activity in infected MS patients and to determine the type of infection (i.e., latent infection, induction of proliferation of infected B cells, lytic replication) that is predominant at different clinical phases of the disease. The integration of these data might eventually allow us to better define the role of EBV in the etiology and pathogenesis of MS and might also generate exciting insights into the immunobiology of host–EBV interactions.

## References

[1] Koch-Henriksen N Magyari M. Apparent changes in the epidemiology and severity of multiple sclerosis, Nature reviews. Neurology. (2021) 17:676–88. doi: 10.1038/s41582-021-00556-y 34584250

[2] Moutsianas L Agarwala V Fuchsberger C Flannick J Rivas MA Gaulton KJ. The power of gene-based rare variant methods to detect disease-associated variation and test hypotheses about complex disease. PLoS Genet. (2015) 11:e1005165. doi: 10.1371/journal.pgen.1005165 25906071 PMC4407972

[3] Lucchinetti C Brck W Parisi J Scheithauer B Rodriguez M Lassmann H. Heterogeneity of multiple sclerosis lesions: Implications for the pathogenesis of demyelination. Ann Neurol. (2000) 47:707–17. doi: 10.1002/1531-8249(200006)47:6<707::aid-ana3>3.0.co;2-q 10852536

[4] Babbe H Roers A Waisman A Lassmann H Goebels N Hohlfeld R. Clonal expansions of CD8(+) T cells dominate the T cell infiltrate in active multiple sclerosis lesions as shown by micromanipulation and single cell polymerase chain reaction. J Exp Med. (2000) 192:393–404. doi: 10.1084/jem.192.3.393 10934227 PMC2193223

[5] Palanichamy A Jahn S Nickles D Derstine M Abounasr A Hauser SL. Rituximab efficiently depletes increased CD20-expressing T cells in multiple sclerosis patients. J Immunol (Baltimore Md.: 1950). (2014) 193:580–6. doi: 10.4049/jimmunol.1400118 PMC4082756 24928997

[6] Obermeier B Mentele R Malotka J Kellermann J Kümpfel T Wekerle H. Matching of oligoclonal immunoglobulin transcriptomes and proteomes of cerebrospinal fluid in multiple sclerosis. Nat Med. (2008) 14:688–93. doi: 10.1038/nm1714 18488038

[7] Attfield KE Jensen LT Kaufmann M Friese MA Fugger L. The immunology of multiple sclerosis, Nature reviews. Immunol 22 734–750 published Online May. (2022) 4:2022. doi: 10.1038/s41577-022-00718-z 35508809

[8] Wekerle H Flügel A Fugger L Schett G Serreze D. Autoimmunity's next top models. Nat Med. (2012) 18:66–70. doi: 10.1038/nm.2635 22227675

[9] Khadilkar SV O Sahni A Agarwal S. A case control study of environmental risk factors in Indians with multiple sclerosis. Neurol Asia. (2005) 10:47–52.

[10] Epstein MA Achong BG Barr YM. Virus particles in cultured lymphoblasts from burkitt's lymphoma. Lancet (London England). (1964) 1:702–3. doi: 10.1016/s0140-6736(64)91524-7 14107961

[11] Weiss LM Strickler JG Warnke RA Purtilo DT Sklar J. Epstein-Barr viral DNA in tissues of Hodgkin's disease. Am J Pathol. (1987) 129:86–91. 2821817 PMC1899692

[12] Tavakoli A Monavari SH Solaymani Mohammadi F Kiani SJ Armat S Farahmand M. Association between Epstein-Barr virus infection and gastric cancer: a systematic review and meta-analysis. BMC Cancer. (2020) 20:493. doi: 10.1186/s12885-020-07013-x 32487043 PMC7268387

[13] Lam WKJ Chan KCA Lo YMD. Plasma Epstein-Barr virus DNA as an archetypal circulating tumour DNA marker. J Pathol. (2019) 247:641–9. doi: 10.1002/path.5249 PMC6594142 30714167

[14] Rickinson AB Yao QY Wallace LE. The Epstein-Barr virus as a model of virus-host interactions. Br Med Bull. (1985) 41:75–9. doi: 10.1093/oxfordjournals.bmb.a072030 2982449

[15] Schwarzmann F Jäger M Hornef M Prang N Wolf H. Epstein-Barr viral gene expression in B-lymphocytes. Leukemia lymphoma. (1998) 30:123–9. doi: 10.3109/10428199809050935 9669682

[16] Amon W Farrell PJ. Reactivation of Epstein-Barr virus from latency. Rev Med Virol. (2005) 15:149–56. doi: 10.1002/rmv.456 15546128

[17] Cohen JI. Epstein-Barr virus infection. New Engl J Med. (2000) 343:481–92. doi: 10.1056/NEJM200008173430707 10944566

[18] Henke CE Kurland LT Elveback LR. Infectious mononucleosis in Rochester, Minnesota, 1950 through 1969. Am J Epidemiol. (1973) 98:483–90. doi: 10.1093/oxfordjournals.aje.a121577 4767624

[19] Rapp CE Hewetson JF. Infectious mononucleosis and the Epstein-Barr virus. Am J Dis Children (1960). (1978) 132:78–86. doi: 10.1001/archpedi.1978.02120260080020 203184

[20] Yao QY Rickinson AB Epstein MA. A re-examination of the Epstein-Barr virus carrier state in healthy seropositive individuals. Int J Cancer. (1985) 35:35–42. doi: 10.1002/ijc.2910350107 2981780

[21] Anagnostopoulos I Hummel M Kreschel C Stein H. Morphology, immunophenotype, and distribution of latently and/or productively Epstein-Barr virus-infected cells in acute infectious mononucleosis: implications for the interindividual infection route of Epstein-Barr virus. Blood. (1995) 85:744–50. doi: 10.1182/blood.V85.3.744.bloodjournal853744 7530505

[22] Allday MJ Crawford DH. Role of epithelium in EBV persistence and pathogenesis of B-cell tumours. Lancet (London England). (1988) 1:855–7. doi: 10.1016/S0140-6736(88)91604-2 2895366

[23] Hutt-Fletcher LM. Epstein-Barr virus entry. J Virol. (2007) 81:7825–32. doi: 10.1128/JVI.00445-07 PMC1951282 17459936

[24] Callan MF Tan L Annels N Ogg GS Wilson JD O'Callaghan CA. Direct visualization of antigen-specific CD8+ T cells during the primary immune response to Epstein-Barr virus *In vivo* . J Exp Med. (1998) 187:1395–402. doi: 10.1084/jem.187.9.1395 PMC2212279 9565632

[25] Chijioke O Müller A Feederle R Barros MHM Krieg C Emmel V. Human natural killer cells prevent infectious mononucleosis features by targeting lytic Epstein-Barr virus infection. Cell Rep. (2013) 5:1489–98. doi: 10.1016/j.celrep.2013.11.041 PMC3895765 24360958

[26] Amarillo ME Moyano A Ferressini Gerpe N de Matteo E Preciado MV Chabay P. Tonsillar cytotoxic CD4 T cells are involved in the control of EBV primary infection in children. Sci Rep. (2024) 14:2135. doi: 10.1038/s41598-024-52666-4 38273012 PMC10810912

[27] Thorley-Lawson DA. EBV persistence–introducing the virus. Curr topics Microbiol Immunol. (2015) 390:151–209. doi: 10.1007/978-3-319-22822-8_8 PMC5125397 26424647

[28] Babcock GJ Hochberg D Thorley-Lawson AD. The expression pattern of Epstein-Barr virus latent genes *in vivo* is dependent upon the differentiation stage of the infected B cell. Immunity. (2000) 13:497–506. doi: 10.1016/s1074-7613(00)00049-2 11070168

[29] Kang M-S Kieff E. Epstein-Barr virus latent genes. Exp Mol Med. (2015) 47:e131. doi: 10.1038/emm.2014.84 25613728 PMC4314583

[30] Thomas OG Rickinson A Palendira U. Epstein-Barr virus and multiple sclerosis: moving from questions of association to questions of mechanism. Clin Trans Immunol. (2023) 12:e1451. doi: 10.1002/cti2.1451 PMC10191779 37206956

[31] Babcock GJ Decker LL Volk M Thorley-Lawson DA. EBV persistence in memory B cells *in vivo* . Immunity. (1998) 9:395–404. doi: 10.1016/s1074-7613(00)80622-6 9768759

[32] Murata T Sato Y Kimura H. Modes of infection and oncogenesis by the Epstein-Barr virus. Rev Med Virol. (2014) 24:242–53. doi: 10.1002/rmv.1786 24578255

[33] Tovey MG Lenoir G Begon-Lours J. Activation of latent Epstein-Barr virus by antibody to human IgM. Nature. (1978) 276:270–2. doi: 10.1038/276270a0 213727

[34] Murata T. Regulation of Epstein-Barr virus reactivation from latency. Microbiol Immunol. (2014) 58:307–17. doi: 10.1111/1348-0421.12155 24786491

[35] Sumaya CV Myers LW Ellison GW. Epstein-Barr virus antibodies in multiple sclerosis. Arch Neurol. (1980) 37:94–6. doi: 10.1001/archneur.1980.00500510052009 6243930

[36] Thacker EL Mirzaei F Ascherio A. Infectious mononucleosis and risk for multiple sclerosis: a meta-analysis. Ann Neurol. (2006) 59:499–503. doi: 10.1002/ana.20820 16502434

[37] Bach J-F. The effect of infections on susceptibility to autoimmune and allergic diseases. New Engl J Med. (2002) 347:911–20. doi: 10.1056/NEJMra020100 12239261

[38] Lünemann JD Münz C. EBV in MS: guilty by association? Trends Immunol. (2009) 30:243–8. doi: 10.1016/j.it.2009.03.007 19428300

[39] Bjornevik K Cortese M Healy BC Kuhle J Mina MJ Leng Y. Longitudinal analysis reveals high prevalence of Epstein-Barr virus associated with multiple sclerosis. Sci (New York N.Y.). (2022) 375:296–301. doi: 10.1126/science.abj8222 35025605

[40] Loosen SH Doege C Meuth SG Luedde T Kostev K Roderburg C. Infectious mononucleosis is associated with an increased incidence of multiple sclerosis: Results from a cohort study of 32,116 outpatients in Germany. Front Immunol. (2022) 13:937583. doi: 10.3389/fimmu.2022.937583 35983044 PMC9379368

[41] Henle W Henle G. Epstein-barr virus (EBV) and immunodeficiencies. In: Szentivanyi A Friedman H, editors. Viruses, Immunity, and Immunodeficiency. Springer US, Boston, MA (1986).

[42] Cusick MF Libbey JE Fujinami RS. Molecular mimicry as a mechanism of autoimmune disease. Clin Rev Allergy Immunol. (2012) 42:102–11. doi: 10.1007/s12016-011-8294-7 PMC3266166 22095454

[43] Liu R Du S Zhao L Jain S Sahay K Rizvanov A. Autoreactive lymphocytes in multiple sclerosis: Pathogenesis and treatment target. Front Immunol. (2022) 13:996469. doi: 10.3389/fimmu.2022.996469 36211343 PMC9539795

[44] Fletcher JM Lalor SJ Sweeney CM Tubridy N Mills KHG. T cells in multiple sclerosis and experimental autoimmune encephalomyelitis. Clin Exp Immunol. (2010) 162:1–11. doi: 10.1111/j.1365-2249.2010.04143.x 20682002 PMC2990924

[45] Bar-Or A Pender MP Khanna R Steinman L Hartung H-P Maniar T. Epstein-barr virus in multiple sclerosis: theory and emerging immunotherapies. Trends Mol Med. (2020) 26:296–310. doi: 10.1016/j.molmed.2019.11.003 31862243 PMC7106557

[46] Goodin D. Genetic and environmental susceptibility to multiple sclerosis. MRAJ. (2021) 9. doi: 10.18103/mra.v9i6.2413 PMC7984655 33750973

[47] Munger KL Levin LI O'Reilly EJ Falk KI Ascherio A. Anti-Epstein-Barr virus antibodies as serological markers of multiple sclerosis: a prospective study among United States military personnel. Multiple sclerosis (Houndmills Basingstoke England). (2011) 17:1185–93. doi: 10.1177/1352458511408991 PMC3179777 21685232

[48] Levin LI Munger KL Rubertone MV Peck CA Lennette ET Spiegelman D. Temporal relationship between elevation of epstein-barr virus antibody titers and initial onset of neurological symptoms in multiple sclerosis. JAMA. (2005) 293:2496–500. doi: 10.1001/jama.293.20.2496 15914750

[49] Ascherio A Munger KL Lennette ET Spiegelman D Hernán MA Olek MJ. Epstein-Barr virus antibodies and risk of multiple sclerosis: a prospective study. JAMA. (2001) 286:3083–8. doi: 10.1001/jama.286.24.3083 11754673

[50] DeLorenze GN Munger KL Lennette ET Orentreich N Vogelman JH Ascherio A. Epstein-Barr virus and multiple sclerosis: evidence of association from a prospective study with long-term follow-up. Arch Neurol. (2006) 63:839–44. doi: 10.1001/archneur.63.6.noc50328 16606758

[51] Walton C King R Rechtman L Kaye W Leray E Marrie RA. Rising prevalence of multiple sclerosis worldwide: Insights from the Atlas of MS, third edition. Multiple sclerosis (Houndmills Basingstoke England). (2020) 26:1816–21. doi: 10.1177/1352458520970841 PMC7720355 33174475

[52] Cohen JI Corey GR. Cytomegalovirus infection in the normal host. Medicine. (1985) 64:100–14. doi: 10.1097/00005792-198503000-00003 2983175

[53] Bray PF Luka J Culp KW Schlight JP. Antibodies against Epstein-Barr nuclear antigen (EBNA) in multiple sclerosis CSF, and two pentapeptide sequence identities between EBNA and myelin basic protein. Neurology. (1992) 42:1798–804. doi: 10.1212/wnl.42.9.1798 1381067

[54] Lünemann JD Jelcić I Roberts S Lutterotti A Tackenberg B Martin R. EBNA1-specific T cells from patients with multiple sclerosis cross react with myelin antigens and co-produce IFN-gamma and IL-2. J Exp Med. (2008) 205:1763–73. doi: 10.1084/jem.20072397 PMC2525578 18663124

[55] ICTV Report Chapters. Virus Taxonomy: The Classification and Nomenclature of Viruses the Online (10th) Report of the ICTV. Available online at: https://ictv.global/report (Accessed December 21, 2023).

[56] Cannon MJ Schmid DS Hyde TB. Review of cytomegalovirus seroprevalence and demographic characteristics associated with infection. Rev Med Virol. (2010) 20:202–13. doi: 10.1002/rmv.655 20564615

[57] Dowd JB Aiello AE Alley DE. Socioeconomic disparities in the seroprevalence of cytomegalovirus infection in the US population: NHANES III. Epidemiol infection. (2009) 137:58–65. doi: 10.1017/S0950268808000551 PMC3806637 18413004

[58] Balfour HH Sifakis F Sliman JA Knight JA Schmeling DO Thomas W. Age-specific prevalence of Epstein-Barr virus infection among individuals aged 6-19 years in the United States and factors affecting its acquisition. J Infect Dis. (2013) 208:1286–93. doi: 10.1093/infdis/jit321 23868878

[59] Narayanan S Shanker A Khera T Subramaniam B. Neurofilament light: a narrative review on biomarker utility. Faculty Rev. (2021) 10:46. doi: 10.12703/r/10-46 PMC8170685 34131656

[60] Cortese M Leng Y Bjornevik K Mitchell M Healy BC Mina MJ. Serologic response to the epstein-barr virus peptidome and the risk for multiple sclerosis. JAMA Neurol. (2024) 81:515–24. doi: 10.1001/jamaneurol.2024.0272 PMC10949154 38497939

[61] Taylor GS Long HM Brooks JM Rickinson AB Hislop AD. The immunology of Epstein-Barr virus-induced disease. Annu Rev Immunol. (2015) 33:787–821. doi: 10.1146/annurev-immunol-032414-112326 25706097

[62] Swain SL McKinstry KK Strutt TM. Expanding roles for CD4⁺ T cells in immunity to viruses, Nature reviews. Immunology. (2012) 12:136–48. doi: 10.1038/nri3152 PMC3764486 22266691

[63] Callan MF Steven N Krausa P Wilson JD Moss PA Gillespie GM. Large clonal expansions of CD8+ T cells in acute infectious mononucleosis. Nat Med. (1996) 2:906–11. doi: 10.1038/nm0896-906 8705861

[64] Long HM Leese AM Chagoury OL Connerty SR Quarcoopome J Quinn LL. Cytotoxic CD4+ T cell responses to EBV contrast with CD8 responses in breadth of lytic cycle antigen choice and in lytic cycle recognition. J Immunol (Baltimore Md.: 1950). (2011) 187:92–101. doi: 10.4049/jimmunol.1100590 PMC3154640 21622860

[65] Levitskaya J Coram M Levitsky V Imreh S Steigerwald-Mullen PM Klein G. Inhibition of antigen processing by the internal repeat region of the Epstein-Barr virus nuclear antigen-1. Nature. (1995) 375:685–8. doi: 10.1038/375685a0 7540727

[66] Paludan C Schmid D Landthaler M Vockerodt M Kube D Tuschl T. Endogenous MHC class II processing of a viral nuclear antigen after autophagy. Sci (New York N.Y.). (2005) 307:593–6. doi: 10.1126/science.1104904 15591165

[67] Wherry EJ. T cell exhaustion. Nat Immunol. (2011) 12:492–9. doi: 10.1038/ni.2035 21739672

[68] Salou M Garcia A Michel L Gainche-Salmon A Loussouarn D Nicol B. Expanded CD8 T-cell sharing between periphery and CNS in multiple sclerosis. Ann Clin Trans Neurol. (2015) 2:609–22. doi: 10.1002/acn3.199 PMC4479522 26125037

[69] Booss J Esiri MM Tourtellotte WW Mason DY. Immunohistological analysis of T lymphocyte subsets in the central nervous system in chronic progressive multiple sclerosis. J neurological Sci. (1983) 62:219–32. doi: 10.1016/0022-510x(83)90201-0 6607973

[70] Lossius A Johansen JN Vartdal F Robins H Jūratė Šaltytė B Holmøy T. High-throughput sequencing of TCR repertoires in multiple sclerosis reveals intrathecal enrichment of EBV-reactive CD8+ T cells. Eur J Immunol. (2014) 44:3439–52. doi: 10.1002/eji.201444662 25103993

[71] Fogdell-Hahn A Ligers A Grønning M Hillert J Olerup O. Multiple sclerosis: a modifying influence of HLA class I genes in an HLA class II associated autoimmune disease. Tissue Antigens. (2000) 55:140–8. doi: 10.1034/j.1399-0039.2000.550205.x 10746785

[72] Ifergan I Kebir H Alvarez JI Marceau G Bernard M Bourbonnière L. Central nervous system recruitment of effector memory CD8+ T lymphocytes during neuroinflammation is dependent on α4 integrin. Brain: J Neurol. (2011) 134:3560–77. doi: 10.1093/brain/awr268 PMC7110084 22058139

[73] Malmeström C Lycke J Haghighi S Andersen O Carlsson L Wadenvik H. Relapses in multiple sclerosis are associated with increased CD8+ T-cell mediated cytotoxicity in CSF. J neuroimmunology. (2008) 196:159–65. doi: 10.1016/j.jneuroim.2008.03.001 18396337

[74] Jacobsen M Cepok S Quak E Happel M Gaber R Ziegler A. Oligoclonal expansion of memory CD8+ T cells in cerebrospinal fluid from multiple sclerosis patients. Brain: J Neurol. (2002) 125:538–50. doi: 10.1093/brain/awf059 11872611

[75] Serafini B Rosicarelli B Franciotta D Magliozzi R Reynolds R Cinque P. Dysregulated Epstein-Barr virus infection in the multiple sclerosis brain. J Exp Med. (2007) 204:2899–912. doi: 10.1084/jem.20071030 PMC2118531 17984305

[76] Vietzen H Berger SM Kühner LM Furlano PL Bsteh G Berger T. Ineffective control of Epstein-Barr-virus-induced autoimmunity increases the risk for multiple sclerosis. Cell. (2023) 186:5705–5718.e13. doi: 10.1016/j.cell.2023.11.015 38091993

[77] Vivier E Tomasello E Baratin M Walzer T Ugolini S. Functions of natural killer cells. Nat Immunol. (2008) 9:503–10. doi: 10.1038/ni1582 18425107

[78] Sollid LM Jabri B. Triggers and drivers of autoimmunity: lessons from coeliac disease, Nature reviews. Immunology. (2013) 13:294–302. doi: 10.1038/nri3407 23493116 PMC3818716

[79] Zhang X Wang T Zhu X Lu Y Li M Huang Z. GMP development and preclinical validation of CAR-T cells targeting a lytic EBV antigen for therapy of EBV-associated Malignancies. Front Immunol. (2023) 14:1103695. doi: 10.3389/fimmu.2023.1103695 36817460 PMC9932894

[80] Torkildsen Ø Myhr K-M Brugger-Synnes P Bjørnevik K. Antiviral therapy with tenofovir in MS. Multiple sclerosis related Disord. (2024) 83:105436. doi: 10.1016/j.msard.2024.105436 38217968

[81] Lünemann JD Edwards N Muraro PA Hayashi S Cohen JI Münz C. Increased frequency and broadened specificity of latent EBV nuclear antigen-1-specific T cells in multiple sclerosis. Brain: J Neurol. (2006) 129:1493–506. doi: 10.1093/brain/awl067 16569670

[82] Lovett-Racke AE Trotter JL Lauber J Perrin PJ June CH Racke MK. Decreased dependence of myelin basic protein-reactive T cells on CD28-mediated costimulation in multiple sclerosis patients. A marker of activated/memory T cells. J Clin Invest. (1998) 101:725–30. doi: 10.1172/JCI1528 PMC508618 9466965

[83] van Noort JM van Sechel AC Bajramovic JJ el Ouagmiri M Polman CH Lassmann H. The small heat-shock protein alpha B-crystallin as candidate autoantigen in multiple sclerosis. Nature. (1995) 375:798–801. doi: 10.1038/375798a0 7596414

[84] Ayoglu B Mitsios N Kockum I Khademi M Zandian A Sjöberg R. Anoctamin 2 identified as an autoimmune target in multiple sclerosis. Proc Natl Acad Sci United States America. (2016) 113:2188–93. doi: 10.1073/pnas.1518553113 PMC4776531 26862169

[85] Wucherpfennig KW Strominger JL. Molecular mimicry in T cell-mediated autoimmunity: viral peptides activate human T cell clones specific for myelin basic protein. Cell. (1995) 80:695–705. doi: 10.1016/0092-8674(95)90348-8 7534214 PMC7133435

[86] Tengvall K Huang J Hellström C Kammer P Biström M Ayoglu B. Molecular mimicry between Anoctamin 2 and Epstein-Barr virus nuclear antigen 1 associates with multiple sclerosis risk. Proc Natl Acad Sci United States America. (2019) 116:16955–60. doi: 10.1073/pnas.1902623116 PMC6708327 31375628

[87] Schneider-Hohendorf T Gerdes LA Pignolet B Gittelman R Ostkamp P Rubelt F. Broader Epstein-Barr virus-specific T cell receptor repertoire in patients with multiple sclerosis. J Exp Med. (2022) 219. doi: 10.1084/jem.20220650 PMC9437111 36048016

[88] Sundqvist E Sundström P Lindén M Hedström AK Aloisi F Hillert J. Epstein-Barr virus and multiple sclerosis: interaction with HLA. Genes Immun. (2012) 13:14–20. doi: 10.1038/gene.2011.42 21776012

[89] Lanz TV Brewer RC Ho PP Moon J-S Jude KM Fernandez D. Clonally expanded B cells in multiple sclerosis bind EBV EBNA1 and GlialCAM. Nature. (2022) 603:321–7. doi: 10.1038/s41586-022-04432-7 PMC9382663 35073561

[90] Thomas OG Bronge M Tengvall K Akpinar B Nilsson OB Holmgren E. Cross-reactive EBNA1 immunity targets alpha-crystallin B and is associated with multiple sclerosis. Sci Adv. (2023) 9:eadg3032. doi: 10.1126/sciadv.adg3032 37196088 PMC10191428

[91] Hauser SL Waubant E Arnold DL Vollmer T Antel J Fox RJ. B-cell depletion with rituximab in relapsing-remitting multiple sclerosis. New Engl J Med. (2008) 358:676–88. doi: 10.1056/NEJMoa0706383 18272891

[92] Thompson AJ Reingold SC Cohen JA. Applying the 2017 McDonald diagnostic criteria for multiple sclerosis - Authors' reply. Lancet Neurol. (2018) 17:499–500. doi: 10.1016/S1474-4422(18)30168-6 29778360

[93] Joseph FG Hirst CL Pickersgill TP Ben-Shlomo Y Robertson NP Scolding NJ. CSF oligoclonal band status informs prognosis in multiple sclerosis: a case control study of 100 patients. J neurology neurosurgery Psychiatry. (2009) 80:292–6. doi: 10.1136/jnnp.2008.150896 18829628

[94] Pender MP. Infection of autoreactive B lymphocytes with EBV, causing chronic autoimmune diseases. Trends Immunol. (2003) 24:584–8. doi: 10.1016/j.it.2003.09.005 14596882

[95] Magliozzi R Serafini B Rosicarelli B Chiappetta G Veroni C Reynolds R. B-cell enrichment and Epstein-Barr virus infection in inflammatory cortical lesions in secondary progressive multiple sclerosis. J neuropathology Exp Neurol. (2013) 72:29–41. doi: 10.1097/NEN.0b013e31827bfc62 23242282

[96] Hassani A Corboy JR Al-Salam S Khan G. Epstein-Barr virus is present in the brain of most cases of multiple sclerosis and may engage more than just B cells. PLoS One. (2018) 13:e0192109. doi: 10.1371/journal.pone.0192109 29394264 PMC5796799

[97] Serafini B Rosicarelli B Magliozzi R Stigliano E Aloisi F. Detection of ectopic B-cell follicles with germinal centers in the meninges of patients with secondary progressive multiple sclerosis. Brain Pathol (Zurich Switzerland). (2004) 14:164–74. doi: 10.1111/j.1750-3639.2004.tb00049.x PMC8095922 15193029

[98] Pender MP Csurhes PA Lenarczyk A Pfluger CMM Burrows SR. Decreased T cell reactivity to Epstein-Barr virus infected lymphoblastoid cell lines in multiple sclerosis. J neurology neurosurgery Psychiatry. (2009) 80:498–505. doi: 10.1136/jnnp.2008.161018 PMC2663364 19015225

[99] Hauser SL Bar-Or A Comi G Giovannoni G Hartung H-P Hemmer B. Ocrelizumab versus interferon beta-1a in relapsing multiple sclerosis. New Engl J Med. (2017) 376:221–34. doi: 10.1056/NEJMoa1601277 28002679

[100] Montalban X Hauser SL Kappos L Arnold DL Bar-Or A Comi G. Ocrelizumab versus placebo in primary progressive multiple sclerosis. New Engl J Med. (2017) 376:209–20. doi: 10.1056/NEJMoa1606468 28002688

[101] Willis SN Stadelmann C Rodig SJ Caron T Gattenloehner S Mallozzi SS. Epstein-Barr virus infection is not a characteristic feature of multiple sclerosis brain. Brain: J Neurol. (2009) 132:3318–28. doi: 10.1093/brain/awp200 PMC2792367 19638446

[102] Peferoen LAN Lamers F Lodder LNR Gerritsen WH Huitinga I Melief J. Epstein Barr virus is not a characteristic feature in the central nervous system in established multiple sclerosis. Brain: J Neurol. (2010) 133:e137. doi: 10.1093/brain/awp296 19917644

[103] Sargsyan SA Shearer AJ Ritchie AM Burgoon MP Anderson S Hemmer B. Absence of Epstein-Barr virus in the brain and CSF of patients with multiple sclerosis. Neurology. (2010) 74:1127–35. doi: 10.1212/WNL.0b013e3181d865a1 PMC2865779 20220124

[104] Meffre E Wardemann H. B-cell tolerance checkpoints in health and autoimmunity. Curr Opin Immunol. (2008) 20:632–8. doi: 10.1016/j.coi.2008.09.001 18848883

[105] Price AM Dai J Bazot Q Patel L Nikitin PA Djavadian R. Epstein-Barr virus ensures B cell survival by uniquely modulating apoptosis at early and late times after infection. eLife. (2017) 6. doi: 10.7554/eLife.22509 PMC5425254 28425914

[106] Mancao C Hammerschmidt W. Epstein-Barr virus latent membrane protein 2A is a B-cell receptor mimic and essential for B-cell survival. Blood. (2007) 110:3715–21. doi: 10.1182/blood-2007-05-090142 PMC2077319 17682125

[107] Caldwell RG Wilson JB Anderson SJ Longnecker R. Epstein-Barr virus LMP2A drives B cell development and survival in the absence of normal B cell receptor signals. Immunity. (1998) 9:405–11. doi: 10.1016/s1074-7613(00)80623-8 9768760

[108] Høglund RA Bremel RD Homan EJ Torsetnes SB Lossius A Holmøy T. CD4+ T cells in the blood of MS patients respond to predicted epitopes from B cell receptors found in spinal fluid. Front Immunol. (2020) 11:598. doi: 10.3389/fimmu.2020.00598 32328067 PMC7160327

[109] Høglund RA Lossius A Johansen JN Homan J Benth JŠ Robins H. In silico prediction analysis of idiotope-driven T-B cell collaboration in multiple sclerosis. Front Immunol. (2017) 8:1255. doi: 10.3389/fimmu.2017.01255 29038659 PMC5630699

[110] Holmøy T Vartdal F Hestvik AL Munthe L Bogen B. The idiotype connection: linking infection and multiple sclerosis. Trends Immunol. (2010) 31:56–62. doi: 10.1016/j.it.2009.11.001 19962346

[111] Soldan SS Su C Monaco MC Yoon L Kannan T Zankharia U. Multiple sclerosis patient-derived spontaneous B cells have distinct EBV and host gene expression profiles in active disease. Nat Microbiol. (2024) 9:1540–54. doi: 10.1038/s41564-024-01699-6 PMC11900839 38806670

[112] Posso-Osorio I Tobón GJ Cañas CA. Human endogenous retroviruses (HERV) and non-HERV viruses incorporated into the human genome and their role in the development of autoimmune diseases. J Trans Autoimmun. (2021) 4:100137. doi: 10.1016/j.jtauto.2021.100137 PMC8669383 34917914

[113] Morandi E Tanasescu R Tarlinton RE Constantinescu CS Zhang W Tench C. The association between human endogenous retroviruses and multiple sclerosis: A systematic review and meta-analysis. PLoS One. (2017) 12:e0172415. doi: 10.1371/journal.pone.0172415 28207850 PMC5313176

[114] Kremer D Gruchot J Weyers V Oldemeier L Göttle P Healy L. pHERV-W envelope protein fuels microglial cell-dependent damage of myelinated axons in multiple sclerosis. Proc Natl Acad Sci United States America. (2019) 116:15216–25. doi: 10.1073/pnas.1901283116 PMC6660731 31213545

[115] Antony JM van Marle G Opii W Butterfield DA Mallet F Yong VW. Human endogenous retrovirus glycoprotein-mediated induction of redox reactants causes oligodendrocyte death and demyelination. Nat Neurosci. (2004) 7:1088–95. doi: 10.1038/nn1319 15452578

[116] Sutkowski N Conrad B Thorley-Lawson DA Huber BT. Epstein-Barr virus transactivates the human endogenous retrovirus HERV-K18 that encodes a superantigen. Immunity. (2001) 15:579–89. doi: 10.1016/s1074-7613(01)00210-2 11672540

[117] Wieland L Schwarz T Engel K Volkmer I Krüger A Tarabuko A. Epstein-barr virus-induced genes and endogenous retroviruses in immortalized B cells from patients with multiple sclerosis. Cells. (2022) 11. doi: 10.3390/cells11223619 PMC9688211 36429047

[118] Flamand L Menezes J. Cyclic AMP-responsive element-dependent activation of Epstein-Barr virus zebra promoter by human herpesvirus 6. J Virol. (1996) 70:1784–91. doi: 10.1128/JVI.70.3.1784-1791.1996 PMC190004 8627701

[119] Gross AJ Hochberg D Rand WM Thorley-Lawson DA. EBV and systemic lupus erythematosus: a new perspective. J Immunol (Baltimore Md.: 1950). (2005) 174:6599–607. doi: 10.4049/jimmunol.174.11.6599 15905498

[120] Evans AS. The spectrum of infections with Epstein-Barr virus: a hypothesis. J Infect Dis. (1971) 124:330–7. doi: 10.1093/infdis/124.3.330 4335371

[121] Madhavan M Nevin ZS Shick HE Garrison E Clarkson-Paredes C Karl M. Induction of myelinating oligodendrocytes in human cortical spheroids. Nat Methods. (2018) 15:700–6. doi: 10.1038/s41592-018-0081-4 PMC6508550 30046099

[122] Matsui TK Matsubayashi M Sakaguchi YM Hayashi RK Zheng C Sugie K. Six-month cultured cerebral organoids from human ES cells contain matured neural cells. Neurosci Lett. (2018) 670:75–82. doi: 10.1016/j.neulet.2018.01.040 29398520

[123] Lancaster MA Knoblich JA. Generation of cerebral organoids from human pluripotent stem cells. Nat Protoc. (2014) 9:2329–40. doi: 10.1038/nprot.2014.158 PMC4160653 25188634

[124] Zdimerova H Murer A Engelmann C Raykova A Deng Y Gujer C. Attenuated immune control of Epstein-Barr virus in humanized mice is associated with the multiple sclerosis risk factor HLA-DR15. Eur J Immunol. (2021) 51:64–75. doi: 10.1002/eji.202048655 32949466

[125] Sundqvist E Bergström T Daialhosein H Nyström M Sundström P Hillert J. Cytomegalovirus seropositivity is negatively associated with multiple sclerosis. Multiple sclerosis (Houndmills Basingstoke England). (2014) 20:165–73. doi: 10.1177/1352458513494489 23999606

[126] Grut V Biström M Salzer J Stridh P Jons D Gustafsson R. Cytomegalovirus seropositivity is associated with reduced risk of multiple sclerosis-a presymptomatic case-control study. Eur J Neurol. (2021) 28:3072–9. doi: 10.1111/ene.14961 34107122

[127] Comabella M Tintore M Sao Avilés A Carbonell-Mirabent P Malhotra S Rovira A. Increased cytomegalovirus immune responses at disease onset are protective in the long-term prognosis of patients with multiple sclerosis. J neurology neurosurgery Psychiatry. (2023) 94:173–80. doi: 10.1136/jnnp-2022-330205 36344261

[128] Zivadinov R Nasuelli D Tommasi MA Serafin M Bratina A Ukmar M. Positivity of cytomegalovirus antibodies predicts a better clinical and radiological outcome in multiple sclerosis patients. Neurological Res. (2006) 28:262–9. doi: 10.1179/016164106X98134 16687051

